# Surgery due to mechanical bowel obstruction in relapsed ovarian cancer: clinical and surgical results of a bicentric analysis of 87 patients

**DOI:** 10.1007/s00404-021-06237-x

**Published:** 2021-10-01

**Authors:** R. Armbrust, R. Chekerov, S. Sander, M. Biebl, S. Chopra, Jonathan Krell, Natasha Rinne, Katherine Nixon, C. Fotopoulou, J. Sehouli

**Affiliations:** 1 Department of Gynecology with Center for Oncological Surgery, Charité-University Hospital Berlin, Augustenburger Platz 1, 13353 Berlin, Germany; 2 Department of Surgery, Charité-University Hospital Berlin, Berlin, Germany; 3 West London Gynecological Cancer Centre, Imperial College NHS Trust, London, UK

**Keywords:** Recurrent ovarian cancer, Salvage surgery, Ileus

## Abstract

**Introduction:**

Mechanical bowel obstruction is a frequent acute and life-threatening event in relapsed ovarian cancer. Salvage surgery after failure of all conservative approaches, resulting in short bowel syndrome (SBS) constitutes a therapeutic dilemma. Our aim was to evaluate patients’ surgical and clinical outcome in these highly palliative situations. Previous, limited, data reported a high morbidity and mortality. However, recent surgical and therapeutical improvements in relapsed ovarian cancer (ROC) offer better identification of patients who might benefit from surgery in an effort to extend the window of opportunity to subsequently offer these patients novel systemic therapeutic approaches.

**Material and methods:**

All subsequent ROC patients between 2012 and 2017 with acute mechanical bowel obstruction who underwent salvage extraperitoneal en bloc intestinal resection were retrospectively identified. Data were collected from two ESGO certified Ovarian Cancer Centers of Excellence (Charité Berlin and Imperial College London) and systematically evaluated regarding surgical and clinical outcomes.

**Results:**

Overall, 87 ROC patients were included in the analysis (median age 56 years, range 24–88), 47% were platinum resistant. High grade serous was the most common histology (76%) while most of the patients (67%) had at least two previous lines of treatment. Mean observed OS was 7.8 months. After salvage surgery, 46% of the patients had a residual small bowel length < 180 cm and 18% > 180 cm resulting in 41% in need of total parental nutrition. In 80% of the patients a permanent stoma was necessary. 30d morbidity and mortality was 74% and 10%, respectively. More than half of the patients were able to receive further courses of chemotherapy after surgery.

**Discussion:**

Salvage surgery for bowel obstruction in ROC patients needs careful consideration and identification of optimal surgical candidates to have the maximal therapeutic benefit. Despite the challenging morbidity profile, most patients managed to proceed to subsequent novel and conventional systemic treatment and so have their window of therapeutic opportunity extended.

## Introduction

Ovarian cancer (OC) is the fifth most common cancer affecting women in Europe. Despite significant innovations and improvements in the past years in surgical and oncological treatment in primary OC, most patients will experience a relapse within the first 2–3 years, which results in a chronic and often palliative treatment situation [1–3]. In ROC several treatment options are available e.g., secondary debulking surgery, chemotherapy alone or in combination with antiangiogenetic agents, PARP Inhibitors or Immunotherapy [4–6]. However, decision making processes in ROC remains very complex and is influenced by many factors: number of previous therapy lines, BRCA status, surgical outcome after primary surgery, symptom burden, side effects and overall tolerance, radiological and biochemical findings [3]. Attributed to the peritoneal dissemination patterns of the disease, acute and chronic bowel obstruction will occur in 35–51% of the patients and is where most patients will eventually fail [7]. Several treatment options are available in this situation ranging from conservative treatment with bowel stimulating agents, endoscopic approaches with stenting especially for the upper GI and percutaneous gastrostomy, to surgery. Due to the multiple challenges that the clinicians are faced, and the high complex profile of the patients, there are no well-defined decision-making algorithms how to manage these situations and to stratify patients to the optimal treatment pathways i.e., conservative vs surgical.

Surgical interventions include various techniques and strategies, such as en- bloc resections of the involved intestinal package with subsequent terminal proximal ileostomy or jejunostomy, attributed to the extensive impact of the peritoneal carcinosis and inflammation on the affected tissue, so that no dissection planes or anastomotic and repair techniques are feasible. Short bowel syndrome (SBS) with subsequent total parenteral nutrition (TPN) is therefore in inevitable some cases [8].

Even though the evidence so far suggests feasibility of salvage surgery in these challenging situations, described morbidity and mortality is high while most studies are monocentric with a small number of patients [8]. Therefore, the aim of our study is to present the largest cohort of patients with bowel obstruction due to ROC operated in two centers of excellence and to evaluate clinical characteristics and outcome.

## Material and methods

The current analysis and data collection was performed in two institutions: The Department of Gynecology with Center for Oncological Surgery, Charité-Berlin and the West London Gynecological Cancer Centre, Imperial College London. Both centers are approved as Ovarian Cancer Center of Excellence by the European Society of Gynecological Oncology (ESGO).

A retrospective evaluation of all ROC cases with acute and chronic bowel obstruction who underwent surgery between February 2012 and December 2017 was performed. Data were collected prospectively via TOC databank and patient files [9]. In every patient, the detailed tumor pattern was intra- operatively assessed by an independent person based on the surgical procedures performed and by a systematic interview of the surgical team. Postoperatively, all histological findings and collected data were entered into a validated documentation system [‘‘Intraoperative Mapping of Ovarian Cancer’’ (IMO)], especially developed for ovarian neoplasms with special focus on the description of the tumor pattern, maximal tumor burden, postoperative tumor residuals and the amount of preoperative ascites. IMO’’ represents a detailed and objective surgical and histopathological documentation system developed and validated to obtain a better and more objective description of the ovarian tumor spread within the abdominal cavity and to define more precisely the histopathological features of the malignancy [10]. Descriptive Analysis was performed identifying patients´ characteristics and surgical outcome parameters. Patients were regularly evaluated at the end of the treatment for evidence of disease recurrence. Clinical examinations, transvaginal and transabdominal ultrasound, CA-125 (when applicable) assays were performed every 3 months. A computed tomography/magnetic resonance imaging scan was ordered if the previously mentioned examinations revealed any pathology. Postoperative Complications were clinically assessed and also standardized documented in the TOC reporting system according to the well-established Clavien–Dindo Classification [11].

### Decision making

Decision to operate was based on clinical presentation, patient’s choice and discussion of all findings and alternative treatment at a multidisciplinary team level. Surgery was not a choice of treatment in patients with poor performance status (ASA > 2, ECOG > 3) and those cardio vascularly unstable that would be unlikely to cope with surgery and anesthesia. Pre-surgical examination included clinical examination, radiologic imaging, biochemical status to identify the site and number of mechanical obstructions as well as the extent of the disease. In case of gastric outlet obstruction, no surgery was performed, but rather endoscopic stenting techniques were preferred. Conservative approach was the treatment of choice in cases of chronic subileus/ileus, with naso- gastric tube, iv treatment to stimulate peristalsis such as metoclopramide and neostigmin, nil by mouth, and often corticosteroids. Decision to proceed to surgery was after failure of all conservative treatments with worsening of the clinical picture and dilated bowel loops in the imaging with transition point. Patients were discussed the risks of the operation, including the risks of stoma formation and short bowel syndrome.

### Statistics

All data are presented as frequency and rate for categorical variables or median and range for continuous variables. Comparisons between patients surviving less than 6 months and patients surviving at least 6 months were performed using Fisher exact test, *W* test, Kendall *T* b or Mann–Whitney *U* test where appropriate. Estimates of median survival and 1 year and 2 year survival rates were calculated using the Kaplan–Meier method. Log-rank tests were used for univariate statistical comparisons. All data were analyzed using IBM SPSS Statistics 19.0 (SPSS Inc, Chicago, III), and *P* < 0.05 was considered statistically significant.

## Results

The final analysis included 87 patients who underwent salvage surgery due to acute and/or chronic mechanical bowel obstruction.

Median patients age was 52 years (22–87). Detailed information on patients’ characteristics are summarized in Table 1.

**Table 1:** Patient and surgical characteristics

Histology		
Serous	76	91.6%
Mucinous	5	6.0%
Other	2	2.3%
Grading		
Low grade	8	33.3
High grade	14	66.7
FIGO stage (FIGO 2014)		
IIa	1	1.9%
IIb	1	1.9%
IIIb	2	3.8%
IIIc	34	64.2%
IV	14	26.4%
IVa	1	1.9%
ECOG		
0	14	20.6%
1	27	39.7%
2	19	27.9%
3	8	11.8%
Ascites		
No	25	56.8
< 500 ml	11	25.0
> 500 ml	8	18.2

All patients were heavily pretreated both on surgical and systemic level. Over two thirds of them (67%) had at least two lines of previous chemotherapy and 69% at least one past surgical procedure, 21% even more than two. The median time between primary diagnosis and the salvage surgery was 26 months (4 weeks to 60 months), in 20.7% the time interval was more than 60 months. Complete macroscopic tumor complete at primary debulking had been achieved in 60.7%. In 26.1% of the patients residual disease was < 10 mm and in 13.2% > 10 mm. Even though tumor reduction was not the primary outcome in the present analysis, complete resection during salvage surgery was achieved in nearly half of the patients (48.5%). Median duration of surgery was 250 min (190–325 min).

**Fig. 1:**
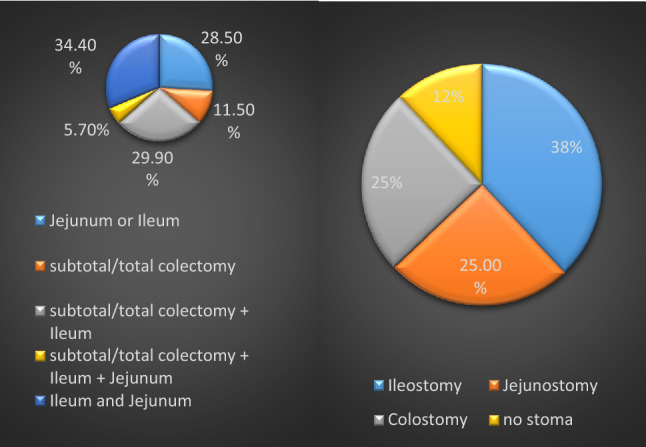
Type of stoma and bowel resection

**Fig. 2:**
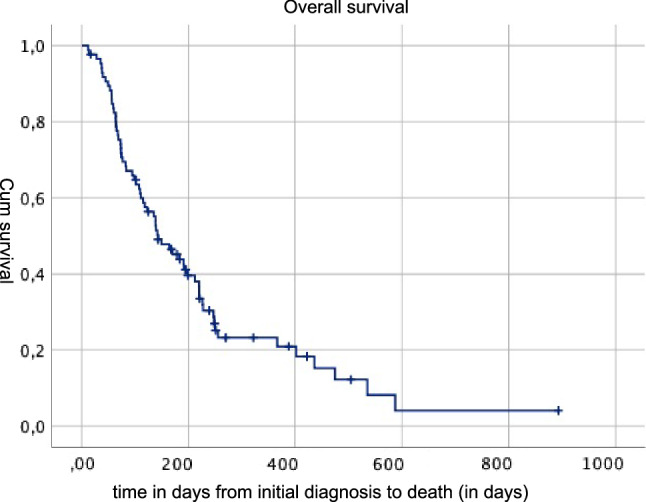
Overall survival in days from initial diagnosis

A majority (65.4%) of patients underwent subtotal or total colectomy, hemicolectomy or rectosigmoid resection. Detailed information can be found in Fig. 1. In 46% of the cases where a small bowel resection was necessary, remaining small bowel length was less than 180 cm. Perioperative antibiotic treatment was given to all the patients until seventh or tenth postoperative day due to common soiling and contamination during the procedures. In 12% of the patients, a primary anastomosis without covering stoma formation was performed. Details about the stoma type are also summarized in Fig. 1. Most of the patients (42%) needed total parenteral nutrition (TPN) postoperatively, this was significantly associated with a residual bowel length < 180 cm. Surgical morbidity rates in the present analysis were similar to those of primary debulking surgery [12] as 26.4% had severe (> Grade III) complications according to Clavien–Dindo classification. Major complications were secondary wound healing (13%), anastomotic leakages in 21%, transfusions (17%) or thromboembolic events. 30d surgical mortality was 10%. Median postoperative OS was 6.4 months (range 4 days–105 months). Multiple logistic regression analysis revealed that platinum sensitivity, ascites less than 500 ml and remaining short bowel length less than 180 cm was significantly associated with prolongation of OS and PFS. To differentiate between patients who might benefit from this type of surgery (more than 6 and more than 18 months) versus those who will have poor outcome (defined as postoperative survival of less than 6 months), three sets of defined parameters were analyzed separately. These consisted of (1) Presurgical patients´ and treatment/ disease related parameters i.e. ECOG, FIGO Stage at primary diagnosis, histological subtype, postoperative residual at primary surgery, platinum free interval. (2) Surgical parameters i.e., residual bowel length, type of bowel resection and type of stoma, number of anastomoses. (3) Postoperative parameters (need of TPN, major complications, subsequent therapy. Regression analysis showed that better ECOG status (1 vs 2 or 3), platinum sensitivity, no or low volume ascites < 500 ml, the type of stoma (colostoma, no ileo- or jejunustoma) number of anastomosis (more than 1), and subsequent therapy were positively associated with the chance of OS of 18 months. Kendall´s tau b coefficient was within 0.35 and 0.5 in this analysis which means a medium efficiency of the correlation coefficient. The weakest correlation was found for ECOG status (tau b = 0.26) whereas the strongest was observed for ascites > 500 ml (tau b = 0.5).Regression analysis also showed that patients with residual postoperative bowel length > 180 cm had 2.4 times higher chance for subsequent therapy but only 0.4 reduced chance when < 180 cm. The residual bowel length, major complications as well as histological subtype did not have a significant impact on survival. Overall, 25 patients were assigned to the group with OS < 18 months and 20 patients in the group with poorer OS of < 6 months, so the groups were relatively small but balanced (Fig. 2).

## Discussion

The present analysis represents the largest so far systematic evaluation of surgical and clinical outcome of heavily pretreated patients with mechanical bowel obstruction who have undergone salvage surgery. We demonstrated that in the majority of the cases, surgery resulted in lifelong TPN due to surgical induced short bowel syndrome. Still, equally the majority of patients were able to proceed to subsequent systemic treatment demonstrating that salvage surgery may potentially extent the window of therapeutic opportunity even this highly palliative setting**.** Conservative treatment can include medical treatment as well as insertion of percutaneous gastric tubes. Usually, Standard conservative management involves fluid and electrolyte resuscitation, nasogastric decompression and fasting, and is most successful (80%) in patients with partial obstruction. While complete obstruction can be managed conservatively in approximately 40% of cases, it incurs a higher rate of bowel resection (30%) for those who fail. he maximum time period of conservative treatment is generally between 3 and 5 days, varying by surgeon, institution and protocol [13]. Another recently published approach could be not only the diagnostic but also the therapeutic use of Gastografin as a hyperosmolar fluid and its use in high concentration. However, the present is somehow inconsistent and also often applies “only” for adhesive bowel obstruction and not for cancer related Ileus [14] However, appropriate management of acute bowel obstruction remains a common clinical challenge [15]. The possibility and feasibility of surgery has been demonstrated in smaller analysis so far [16]. These surgeries should ideally be performed only in a multidisciplinary setting with adequate infrastructure and possibility of home care support Therefore, surgery still remains not being the standard procedure. However, our analyses in 86 cases could show that despite the very high risk for SBS and the need for TPN surgical morbidity rates remain low as well as mortality.

As the overall prognosis still remains poor, we tried to identify patients who might benefit more from this type of surgery and compared a group with poorer OS and better OS (6 vs. 18 months). Highly interesting are the results of the regression analysis: According to our data a better ECOG Status, platinum sensitivity, ascites < 500 ml, the type of stoma and the number of anastomosis were positively associated with the chance of OS of 18 months respectively of more than six months. More interestingly, the residual bowl length and the occurrence of severe postoperative complications had no significant impact. These findings also highlight the importance of presurgical examination structured frailty assessment and the right patient selection which is an emerging concept in surgical approach in OC patients [17]. Even if there not sufficient time or capacity to prepare the patient within a prehabilitation program [17] for surgery, presurgical assessment and structured evaluation also of conservative treatment alternatives methods and multiprofessional support should be key elements in the management of ovarian cancer patients with bowel obstruction. Furthermore the recently presented results of the DESKTOP III trial could demonstrate a meaningful survival benefit (OS, PFS and TFST) in ROC if pts with first platinum sensitive relapse and selected by a positive AGO-Score were applicable [18]. Therefore, our data might also be important prospectively in the decision-making process but should examined further in multicentric trials with focus in patient reported outcomes and survival measures.

### Limitations

Despite being the largest published data so far in ROC patients the study has some significant limitations. It should be highlighted that only patients in stable clinical conditions were enrolled for surgery, especially the absence of bowel perforation, sepsis or fistula. Also, ECOG status had to be 0 or 1 as well as a good ASA status. Obviously, the number of patients in this study remains low and therefore the small number might reduce the power of the results and should be taken in account when interpreting the results. However, it should also be mentioned that both involved centers in this trial were of very high expert in OC and also high volume so the results cannot be equally translated to all institutions.

## Conclusion

Palliative salvage surgery in ROC patients with acute or chronic bowel obstruction is a feasible method with high but acceptable morbidity and mortality rates. However, presurgical patient selection and individual disease history remains crucial as surgery results in SBS and the subsequent need for TPN.

## Data Availability

Data is available and full transparency is given.

## Code availability

Not relevant.
