# Correction to: Surgery due to mechanical bowel obstruction in relapsed ovarian cancer: clinical and surgical results of a bicentric analysis of 87 patients

**DOI:** 10.1007/s00404-021-06322-1

**Published:** 2021-11-22

**Authors:** R. Armbrust, R. Chekerov, S. Sander, M. Biebl, S. Chopra, Jonathan Krell, Natasha Rinne, Katherine Nixon, C. Fotopoulou, J. Sehouli

**Affiliations:** 1 Department of Gynecology with Center for Oncological Surgery, Charité-University Hospital Berlin, Augustenburger Platz 1, 13353 Berlin, Germany; 2 Department of Surgery, Charité-University Hospital Berlin, Berlin, Germany; 3 West London Gynecological Cancer Centre, Imperial College NHS Trust, London, UK

## Correction to: Archives of Gynecology and Obstetrics 10.1007/s00404-021-06237-x

In the original article published, the Fig. 2 is published incorrectly. The correct figure is given below.

**Fig. 2:**
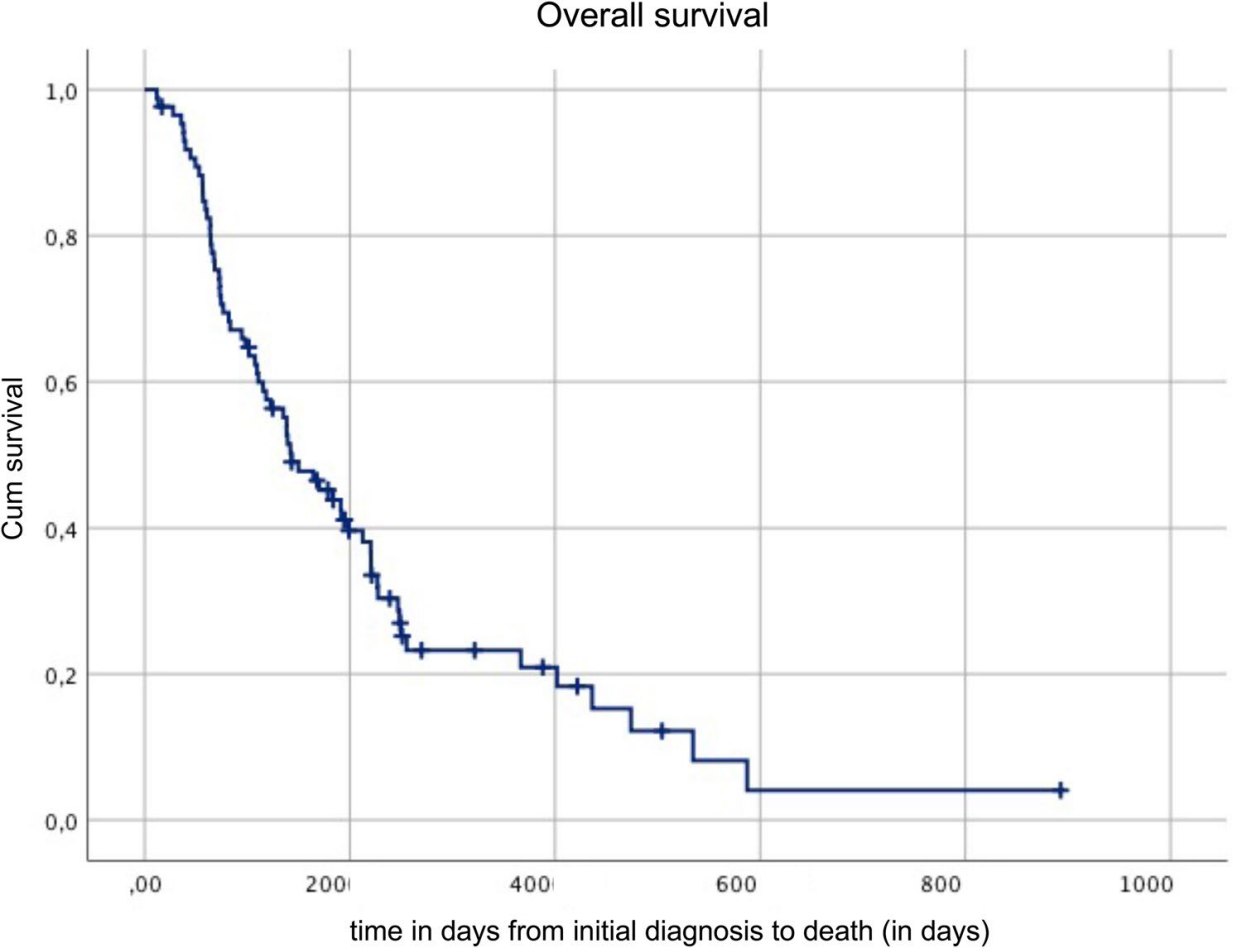
Overall survival in days from initial diagnosis

The original article has been corrected.

